# Targeting DNAJC19 overcomes tumor growth and lung metastasis in NSCLC by regulating PI3K/AKT signaling

**DOI:** 10.1186/s12935-021-02054-z

**Published:** 2021-07-03

**Authors:** Ji Zhou, Yang Peng, Ying-chun Gao, Tai-yu Chen, Peng-cheng Li, Ke Xu, Tao Liu, Tao Ren

**Affiliations:** 1 Health Management Centre, Clinical Medical College and The First Affiliated Hospital of Chengdu Medical College, 278 Baoguang St, Xindu Distr, Chengdu, 610500 Sichuan China; 2 Hematology Department, Clinical Medical College and The First Affiliated Hospital of Chengdu Medical College, Chengdu, 610500 China; 3 Oncology Department, Pengzhou People’s Hospital, Chengdu, 611900 China; 4 Clinical Medical College of Chengdu Medical College, Chengdu, 610500 China; 5 Oncology Department, Clinical Medical College and The First Affiliated Hospital of Chengdu Medical College, 278 Baoguang St, XinduDistr, Chengdu, 610500 Sichuan China

**Keywords:** DNAJC19, Targeted therapy, AKT, Non-small-cell lung cancer, Metastasis

## Abstract

**Background:**

Some driver oncogenes are still unknown in non-small-cell lung cancer (NSCLC). DNAJC19, a major component of the translocation machinery of mitochondrial membranes, is a disease-associated protein. Herein, we report the role of DNAJC19 in NSCLC cell growth and metastasis.

**Methods:**

Immunohistochemistry (IHC) was performed to investigate DNAJC19 expression in NSCLC clinical samples. For knockdown or overexpression assays in A549 or NCI-H1299 lung cancer cells, lentiviral vectors were used. After assessment of cell functions, DNAJC19-knockdown A549 cells were further applied to establish mouse xenograft and metastasis tumor models. Assessments based on the RNA-seq data, western blotting, PCR and IHC were performed for the mechanistic study.

**Results:**

Expression of DNAJC19 was higher in tumors than in noncancerous adjacent tissues. Survival analysis indicated that low DNAJC19 levels were correlated with an increased progression-free survival rate. ShRNA-mediated knockdown of DNAJC19 markedly inhibited cell growth, viability, migration and invasion. Moreover, RNA-seq analysis revealed that the PI3K/AKT signaling pathway was involved in molecular events when A549 cells were treated with shDNAJC19. In addition, DNAJC19 knockdown decreased PI3Kp85a, AKT and p-AKT expression in A549 cells, and cellular functions were greatly rescued in DNAJC19-knockdown A549 cells by ectopic overexpression of AKT. Furthermore, tumor xenograft growth and lung metastasis were markedly repressed in the shDNAJC19 group compared to the control group. As expected, the expression levels of DNAJC19, PI3K and AKT in xenograft mouse samples were also lower in the shDNAJC19 group than in the shCtrl group.

**Conclusions:**

DNAJC19 greatly promotes NSCLC cell growth and lung metastasis by regulating PI3K/AKT signaling, providing a novel therapeutic target for treating NSCLC patients.

**Supplementary Information:**

The online version contains supplementary material available at 10.1186/s12935-021-02054-z.

## Background

Non-small-cell lung cancer (NSCLC) has the highest cancer incidence worldwide [1, 2]. With the adoption of sophisticated surgical and radiotherapeutic modalities, especially tyrosine kinase inhibitors (TKIs) [2, 3] and programmed death-1/programmed death-ligand 1 (PD-1/PD-L1) inhibitors [4–6], overall survival has been increased for NSCLC patients. However, compared to that in other cancer patients, the survival time in most NSCLC patients is relatively short because of adverse outcomes, such as metastasis and relapse [7]. A deeper understanding of the biological traits of NSCLC is required to improve the therapeutic efficacy against tumor cells.

DNAJC19 is a homologous protein of yeast Pam18/Tim14 (yPam18), a component of the mitochondrial protein import machinery in the inner mitochondrial membrane, which is thought to be involved in the ATP-dependent transport of transit proteins from the inner cell membrane to the mitochondrial matrix. It has been reported that DNAJC19 interacts with the mitochondrial prohibit in complex, which disrupts the functional integrity of mitochondria by disturbing phospholipid homeostasis, leading to mitochondrial cristae alterations. In addition, mutations in DNAJC19 have been linked to clinical manifestations, including failure to thrive, ataxia, dilated cardiomyopathy, cryptorchidism, and even hypospadias. Mitochondrial dysfunction, a common phenotypic characteristic of tumor cells, is thought to contribute to the development and progression of various cancers [8]. This raises the possibility that overexpression or mutation of DNAJC19 is associated with tumor development and progression. However, the oncogenic role of DNAJC19 and the associated underlying mechanisms in lung cancer are still far from elucidated.

Recently, the identification of additional driver oncogenes, such as epidermal growth factor receptor (*EGFR*), anaplastic lymphoma kinase (ALK), ROS proto-oncogene 1 (ROS1), and rearranged during transfection (*RET*), in NSCLC patients has led to new targeted treatments [9], greatly improving clinical practice. However, almost 8–40% of driver oncogenes are still unknown in NSCLC [9, 10].

Our data show that DNAJC19 is overexpressed in NSCLC tumor tissues compared to normal tissues. Moreover, despite no difference in overall survival (OS), NSCLC patients with high DNAJC19 expression have poorer progression-free survival (PFS). Furthermore, we used A549 and NCI-H1299 NSCLC cell lines in vitro to investigate the biofunctions of DNAJC19 in cell processes, including cell growth, proliferation, migration, invasion. Interestingly, we found that DNAJC19 increased tumor metastasis and affected xenograft tumor growth in a nude mouse model. More importantly, we explored the underlying regulatory mechanism and report for the first time that the DNAJC19/PI3K/AKT signaling pathway is involved in NSCLC cell proliferation and lung migration.

## Materials and methods

### Human specimens and cell culture

The collection and use of human samples and animal experiments were approved by the ethical review committees of the First Affiliated Hospital of Chengdu Medical College (No. 2020CYFYIRB-BA-89).

Human specimens were collected from 39 NSCLC patients during surgery at the First Affiliated Hospital, Chengdu Medical College. The patient characteristics are summarized in Table 1.

**Table 1:** Correlations between clinicopathological characteristics and DNAJC19 in NSCLC patients

Parameter	DNAJC19 expression		χ^2^	*P*
	Low	High		
Sex				
Male	15	8	0.0307	0.8619
Female	10	6		
Age				
< 60	9	7	0.7270	0.3939
≥ 60	16	7		
Type				
Adenocarcinoma	16	9	0.0003	0.9858
Squamous carcinoma	9	5		
Staging				
T1c–2b	18	10	0.0015	0.9697
T3–4	7	4		
N0	19	8	1.4980	0.2210
N1–2	6	6		
Tissue				
Carcinoma tissue	25	14	4.4860	0.0342
Paracarcinoma tissue	18	2		

The human lung cancer cell lines A549, NCI-H1299,Hela, and SiHa, were obtained from American Type Culture Collection (Manassas, VA, USA), and NCI-H1975, 95-D and MRC-5 were obtained from Cell Bank of the Chinese Academy of Sciences (Shanghai, People’s Republic of China) and cultured in RPMI-1640 medium (Corning, NY, USA) supplemented with 10% heat-inactivated FBS. The cells were maintained at 37 °C in a 5% CO2/95% air humidified incubator.

### Cell transfection

Cell transfections were performed using lentiviral vectors (Shanghai GeneChem Co., Ltd.). DNAJC19, AKT and MYC expression vectors and small hairpin RNAs (shRNAs) targeting DNAJC19 were purchased from GeneChem Co. (Shanghai, China). The DNAJC19 shRNA target sequence was as follows: TTTGCAGGCCGTTACGTTT, and the control sequence was TTCTCCGAACGTGTCACGT.

### Celigo assay, MTT assay and flow cytometric analysis

The indicated cells were plated in a 96-well plate (Corning, NY, USA) at a density of 2,000 cells/well. After 24 h of cell seeding, Celigo (Nexcelom, Massachusetts, USA) was used to count the cell numbers daily for 5 days [11].

The indicated cells were plated in a 96-well plate at a density of 2,000 cells/well. MTT methods were used to assess cell viability at 24, 48, 72, 96 and 120 h after cell seeding (Shanghai Dimgguo, China) according to the manufacturer’s instructions [11].

Apoptotic cells were detected using flow cytometric analysis with the Annexin V-APC kit (eBioscience, ThermoFisher, Shanghai, China) [12] according to the manufacturer’s instructions.

### RNA extraction and qPCR

Total RNA was extracted using TRIzol reagent (Shanghai Pufei Biotech Co., Ltd. Shanghai, China) according to the manufacturer’s instructions. Reverse transcription (RT) and PCR were performed with a High-Capacity cDNA Reverse Transcription kit and a QuantiTect SYBR Green PCR kit (Qiagen, Shanghai, China), respectively. The primer sequences (Shanghai GeneChem Co., Ltd.) were as follows: DNAJC19 forward, 5′-ACAAAACGGGAAGCAGCATTA-3′ and reverse, 5′-AGGAGATCCTCCTTTGTCAGG-3′ and GAPDH forward, 5′-TGACTTCAACAGCGACACCCA-3′ and reverse, 5′-CACCCTGTTGCTGTAGCCAAA-3′.

### Cell migration and invasion

Cell migration was investigated by a Transwell (8-μm pore size; Corning, NY, USA) assay [13]. In brief, A549 and NCI-H1299 cells suspended in serum-free medium were seeded into the upper chambers, and the lower chambers were filled with RPMI-1640 supplemented with 30% FBS. After 16 h of incubation at 37 °C, the cells were fixed and stained.

And the invasion assays [14] were carried out via using Matriel (Corning, NY, USA), which was moved into the upper chambers and incubated 2 h in 37 °C incubator. A549 and NCI-H1299 cells suspended in serum-free medium were seeded into the upper chambers, and the lower chambers were filled with RPMI-1640 supplemented with 30% FBS. After 30 h of incubation at 37 °C, the cells were fixed and stained.

The number of migrated cells was counted using an inverted microscope (CKX41, Olympus, Japan)(200X, 9 fields/chamber). And the assays were triplicate.

### Western blot and immunohistochemistry assays

Western blotting and immunohistochemistry (IHC) were performed as previously described [15]. Antibodies against DNAJC19, PI3Kp85a, p-AKT(T308), AKT, GAPDH, CDH2, MMP2, TWIST, MYC, Caspase 3 and p-mTOR were purchased from Abcam (Cambridge, MA, USA. The catalog is ab230187, ab191606, ab8933, ab18785, ab9485, ab18203, ab37150, ab50887, ab32072, ab32351 and ab109268, respectively), and antibodies against CDH1, ERK1/2, p-P38, P38, MMP9, Snail, mTOR, NFkB-p65, p-ERK1/2, VIM, p-NFkB-p65, p-β-Catenin, β-Catenin, Slug were bought from CST (Massachusetts, USA. The catalog is 14472S, #9107, #4631, #8690, 13667s, 3879s, #2972, #8242, #4376, 3932s, 3033s, 2009s, 9562s, #9585, respectively). And antibody against FN1 was purchased for R&D (Minnesota, USA. The catalog is MAB19182). The antibodies against PARP1, Cytochrome C were bought from ZEN BIO (Chengdu, China. The catalog is 385279, 200,758, respectively).

### RNA-seq analysis

RNA was extracted following the TRIzol reagent manual. RNA was precipitated in 1:1 isopropanol (v/v) and 1 μL glycogen at − 20 °C overnight. Sequencing libraries were generated using NEBNext® UltraTM RNA Library Prep Kit for Illumina® (New England BioLabs, Ltd., USA) following the manufacturer’s recommendations [16, 17]. After adenylation of the 3’ ends of the DNA fragments, the NEBNext Adaptor with a hairpin loop structure was ligated to the fragments to prepare for hybridization. To select cDNA fragments of preferentially 250–300 bp in length, the library fragments were purified with AMPure XP system (Beckman Coulter, Beverly, USA). Then, 3 μL USER Enzyme (NEB, USA) was used with size-selected, adaptor-ligated cDNA at 37 °C for 15 min followed by 5 min at 95 °C before PCR. PCR was performed with Phusion High-Fidelity DNA polymerase, Universal PCR primers and Index (X) Primer. The PCR products were purified (AMPure XP system), and library quality was assessed on the Agilent Bioanalyzer 2100 system.

The clustering of the index-coded samples was performed on a cBot Cluster Generation System using TruSeq PE Cluster Kit v3-cBot-HS (Illumia) according to the manufacturer’s instructions. After cluster generation, the library preparations were sequenced on an Illumina Novaseq platform, and 150 bp paired-end reads were generated. Gene Ontology (GO) and Kyoto Encyclopedia of Genes and Genomes (KEGG) pathway analyses were conducted by clusterProfiler (version 3.4.4).

### Animal experiments

The animal experiments were approved by the local ethical review committees (No. 2020CYFYIRB-BA-89).

Animal experiments were conducted using 6-week-old female Balb/c nude mice [18]. A total of 1 × 10^7^ DNAJC19-knockdown A549 cells or negative control A549 cells were suspended in 100 µl of phosphate-buffered saline and subcutaneously injected into each mouse to establish the xenograft model; every group had 10 mice. After the mice were euthanized via using intraperitoneal of 2% sodium pentobarbital about 70 s, xenograft tumors were dissected and then weighed. The samples were split, and one part was formalin-fixed and paraffin-embedded, while the other was stored in liquid nitrogen.

For the tumor metastasis model [19], mice were injected via the tail vein with DNAJC19-knockdown or negative control A549 cells (2 × 10^6^) (n = 5 per group). After 32 days, an in vivo imaging system for small animals (Lumina LT, Perkin Elmer) was used to assess the metastatic tumors. The animals were anesthetized with isoflurane during the scanning period.

### Statistics

The data are expressed as the mean ± standard deviation (SD). One-way analysis of variance (ANOVA) followed by Tukey’s multiple comparison procedure was used for comparisons of multiple groups. A value of P < 0.05 was considered to be statistically significant. The assays were performed at least three times independently.

## Results

### DNAJC19 is highly expressed in NSCLC tumor tissue

**Fig. 1:**
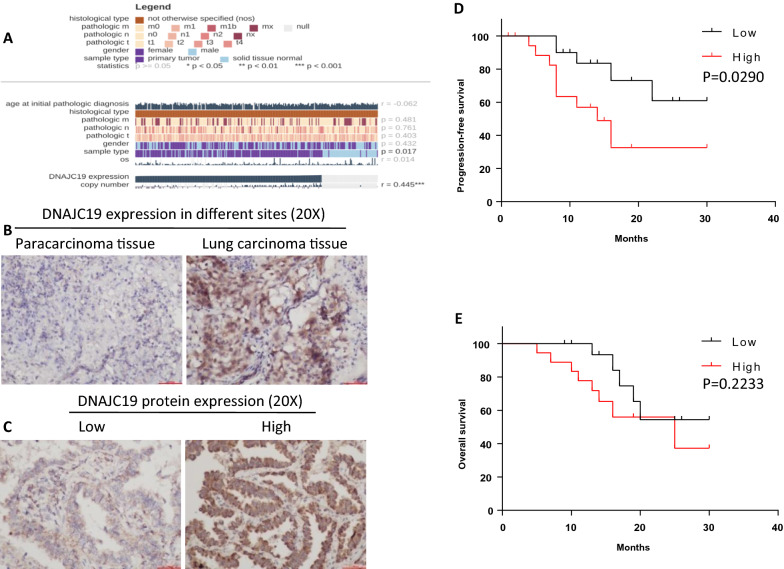
DNAJC19 expression in different sites and its effect on the survival of NSCLC patients. **A** The relationship between DNAJC19 expression and clinical characteristics in NSCLC patients from the MEXPRESS data base. **B** Representative IHC images for difference of DNAJC19 expressions between paracarcinoma tissue and lung cancer tissue, respectively. **C** Representative IHC images for different DNAJC19 expressions in NSCLC samples. **D**, **E** Survival analysis using the Kaplan–Meier method. The patients with high expression of DNAJC19 had longer PFS, not OS, compared with low-expression patients

First, we analyzed the relationship between DNAJC19 expression and clinical characteristics in NSCLC patients using the MEXPRESS data base. DNAJC19 expression was higher in primary tumors than in normal tissues (Fig. 1A). IHC was performed to evaluate DNAJC19 expression in 39 NSCLC samples. As expected, compared to noncancerous adjacent tissue, cancer tissue showed significant overexpression of DNAJC19 (Fig. 1B and Table 1). In addition, the DNAJC19 expression level was diverse in different cancer cases (Fig. 1C). Next, we analyzed the correlation between DNAJC19 expression and clinicopathologic characteristics. As shown in Table 1, no significant differences, except for in the tissue category, were found between the low and high DNAJC19 expression groups in clinical parameters, including sex, age, T staging, N staging, and tumor type. Furthermore, we carried out survival analysis using the Kaplan–Meier method. A low level of DNAJC19 in NSCLC patients was correlated with increased progression-free survival but not overall survival. These data suggest that DNAJC19 expression in NSCLC patients is high and is associated with poor prognosis.

### *Inhibition of DNAJC19 with shRNA decreases NSCLC cell growth, viability, and migration ability *in vitro

The clinical data indicated that DNAJC19 overexpression may be associated with poor prognosis in NSCLC patients. Therefore, we carried out in vitro experiments to investigate whether and how the DNAJC19 protein plays a role in lung cancer cells.

First, we assessed the protein expression of DNAJC19 in different lung cancer cells. As shown in Additional file 1: Figure S1A, the level of DNAJC19 was generally high in A549, 95-D, NCI-H1975 and NCI-H1299 cells. Among these, A549 cells had the lowest level, and NCI-H1299 cells had a relatively higher level of DNAJC19 protein expression and were therefore used in subsequent assays.

**Fig. 2:**
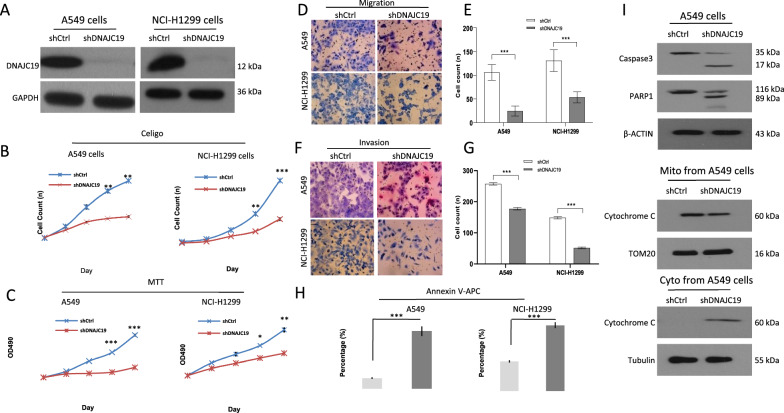
The inhibition of cell growth, viability, apoptosis, migration and invasion abilities in A549 and NCI-H1299 NSCLC cells treated with shDNAJC19 in vitro. **A** Designed shRNA significantly inhibits DNAJC19 protein expression in both lung cancer cells. **B** ShDNAJC19 markedly decreases the cell growth through Celigo. **C** ShDNAJC19 obviously inhibits the cell viability through MTT. **D**, **E** Representative images and semiquantitative analysis of cell migration, and shDNAJC19 markedly decreases the cell migration ability. **F**, **G** Representative images and semiquantitative analysis of cell invasion. ShDNAJC19 markedly decreases the cell invasion ability. **H** ShDNAJC19 significantly increases the cell apoptosis using Annexin V-APC kit. **I** Apoptotic related protein expressions in A549 cells with treatment of DNAJC19 shRNA using western blotting. The data were described as mean ± SD. *P < 0.05, **P < 0.01,***P < 0.001, as compared with the indicated group

We designed shRNAs targeting DNAJC19 (GenBank Accession No. NM_145261) through the online tool provided by BLOCK-iT RNAi Designer (http://rnaidesigner.lifetechnologies.com/rnaiexpress/design.do). The target sequence TTTGCAGGCCGTTACGTTT and control sequence TTCTCCGAACGTGTCACGT were used to design oligonucleotides, which were synthesized by Shanghai Generay Biotech Co., Ltd. After oligonucleotide annealing, we successfully constructed a recombinant lentiviral vector expressing shRNA against the human DNAJC19 gene (Lv-shRNA-DNAJC19) by using the linearized vector GV115 (Shanghai GeneChem Co., Ltd. Shanghai, China) (Additional file 1: Figure S1B–D). Next, we treated A549 and NCI-H1299 cells with DNAJC19 shRNA (shDNAJC19) or control shRNA (shCtrl) (Additional file 1: Figures S1E and Fig. 2A) to investigate cell growth, viability, and migration. As shown in Fig. 2B, A549 and NCI-H1299 cell growth in the shDNAJC19-treated group was markedly inhibited compared with that in the shCtrl group at days 4 and 5, respectively, as determined by the Celigo assay. Compared with the shCtrl group, the shDNAJC19 group had markedly decreased A549 and NCI-H1299 cell viability at days 4 and 5, respectively, as demonstrated by the MTT assay (Fig. 2C). We also found that A549 and NCI-H1299 cell migration and invasion were largely impeded in the shDNAJC19 group compared with the shCtrl group based on migration and invasion assays (Fig. 2D–G). The numbers of migrating cells in the shCtrl and shDNAJC19 groups were 144.3 ± 4.0 vs. 61.7 ± 3.1, respectively, for A549 cells and 235.7 ± 4.5 vs. 115.3 ± 3.5, respectively, for NCI-H1299 cells (Fig. 2D, E). The numbers of invading cells in the shCtrl and shDNAJC19 groups were 256.3 ± 4.0 vs. 148.0 ± 3.5, respectively, for A549 cells and 177.4 ± 4.0 vs 51.0 ± 3.0, respectively, for NCI-H1299 cells (Fig. 2F, G). The Annexin V-APC assay demonstrated that apoptosis of A549 and NCI-H1299 cells was clearly increased in the shDNAJC19 group compared with the shCtrl group (Fig. 2H). And we further used Western blotting to investigate the expression of apoptotic proteins from A549 cells treated with shDNAJC19, as shown in Fig. 2I, either caspase3 or parp1 was largely increased in the shDNAJC19 group compared with the shCtrl group; and the cytochrome C protein was decreased in mitochondria but increased in cytoplasm. Taken together, these results suggest that shRNA-mediated inhibition of DNAJC19 impairs cell growth, viability, migration and invasion in lung cancer cell lines.

### PI3K/AKT is involved in molecular events of DNAJC19 shRNA treatment based on RNA-seq analysis

**Fig. 3:**
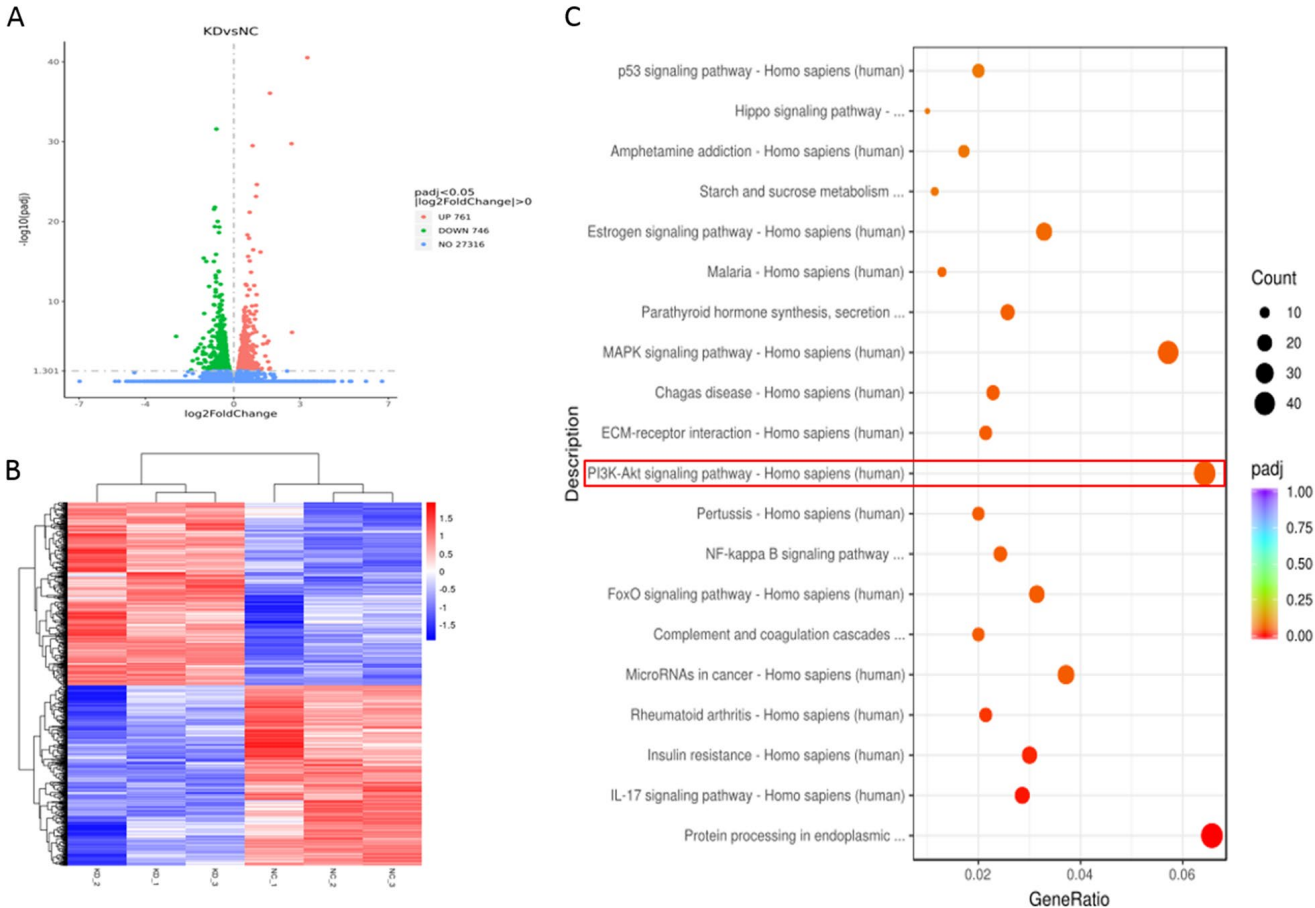
RNA-seq data from A549 cells treated with shDNAJC19 or shRNA control. **A** Volcano plot of mRNA expression in A549 cells treated with shDNAJC19 or shCtrl. **B** Heatmap for each sample. **C** Bubble plot of top 20 enrichment pathways, including the PI3K/AKT pathway (Marked as the red frame)

From the RNA-seq data of Fig. 5, there were 761 upregulated and 746 downregulated proteins based on an analysis of the RNA transcriptomes of 3 A549 samples treated with shDNAJC19 and 3 controls (Fig. 3A). Unsupervised hierarchical clustering distinguished shDNAJC19 from the control with minor overlap (Fig. 3B). The top enriched KEGG pathways were protein processing in endoplasmic, PI3K/AKT signaling pathway, MAPK signaling pathway, and microRNAs in cancer (Fig. 3C). These findings suggested that PI3K/AKT signaling is involved in the molecular events in A549 cells treated with shDNAJC19.

### DNAJC19 directly regulates PI3K/AKT signaling

**Fig. 4:**
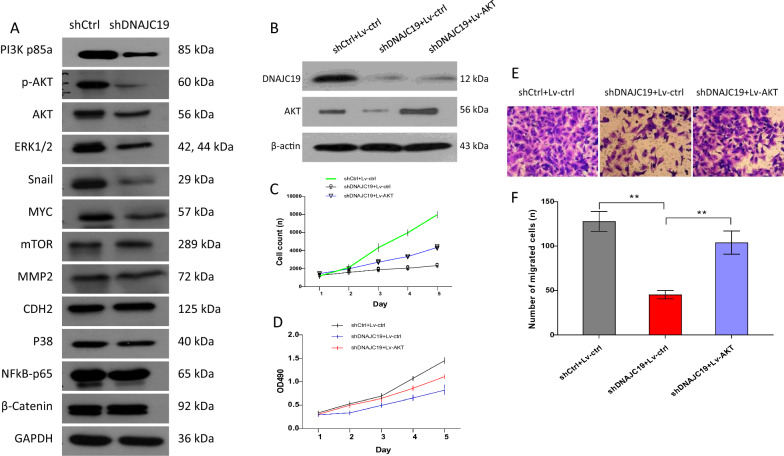
PI3K/AKT pathway is regulated by DNAJC19. **A** The related proteins were investigated in A549 cells treated with shDNAJC19 or shRNA control through western blotting. **B** Lentiviral-mediated AKT (Lv-AKT) overexpression in A549 cells with treatment of DNAJC19 shRNA using western blotting. **C** The growth was strongly restored by overexpression of AKT in A549 cells concurrently treated with shDNAJC19 via Celigo assay. **D** The cell viability was rescued by AKT overexpression in A549 cells with the concurrent treatment with shDNAJC19 using MTT assay. **E**, **F** The migration ability of A549 cells was strongly enhanced by simultaneous AKT overexpression and DNAJC19 knockdown. Data are shown as mean ± SD. **P < 0.01, when in contrast with the indicated group

Next, we performed a western blot assay to investigate a panel of related proteins, including m-TOR, MMP2, CDH2, p38, NFkB-P65, catenin, ERK1/2, MYC, PI3Kp85a, AKT and pAKT(T308), in A549 cells. Interestingly, we found that the protein levels of PI3K, AKT and p-AKT were markedly decreased in A549 cells treated with shDNAJC19 (Fig. 4A). PI3K/AKT signaling plays a key role in tumor cell growth, viability, proliferation, migration and invasion [20]. Next, we overexpressed AKT (Lv-AKT) in A549 lung cancer cells treated with DNAJC19 shRNA (Fig. 4B). As shown in Fig. 4C, the Celigo assay revealed that the growth ability of DNAJC19 shRNA-treated A549 cells compared with DNAJC19 shRNA-knockdown cells was largely restored when Lv-AKT was administered. These data suggest that AKT is regulated by DNAJC19 in lung cancer cells. We thus further tested cell viability by the MTT assay and migration of A549 cells treated with DNAJC19 shRNA. As expected, A549 cell viability was visibly enhanced in the DNAJC19 shRNA plus Lv-AKT group compared with the DNAJC19 shRNA-only group (Fig. 4D). The migration ability of A549 cells was also substantially rescued and further promoted in the combination group of shDNAJC19 plus Lv-AKT compared with the shDNAJC19-only group (Fig. 4E, F). Taken together, these results suggest that DNAJC19 regulates A549 cells probably through modulating PI3K/AKT.

### DNAJC19 has a positive role in xenograft tumor growth and tumor lung metastasis

**Fig. 5:**
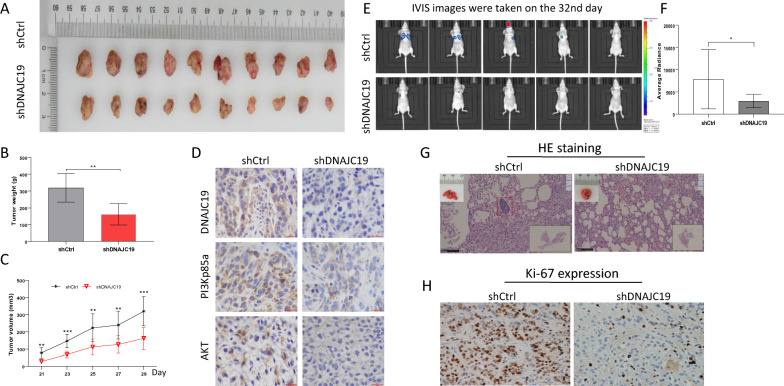
shDNAJC19 significantly decreased tumor growth and metastasis of NSCLC in Balb/c nude mice. **A** Xenograft samples from A549 cells with transfection of shDNAJC19 or shRNA control. **B** The weight of xenograft tumor from A549 cells with transfection of shDNAJC19 or shRNA control. **C** Tumor volume of xenografts from A549 cells with transfection of shDNAJC19 or shRNA control. **D** Immunohistochemistry results for DNAJC19, PI3K and AKT expressions in xenograft tissues, respectively. **E**, **F** Tumor metastasis model from IVIS images and semiquantitative analysis. ShDNAJC19 had fewer average metastatic tumor lesions in each mouse and fewer mice with metastasis were observed in the shDNAJC19 group than in the shCtrl group. **G** The representative photos for the metastasis lesion using HE staining, and the lesion was definitely observed in lung tissue in shControl group (red rectangle). **H** The ki-67 expressions in metastasis lesions are strongly high in shControl group than in shDNAJC19 group using immunohistochemistry. Data are shown as mean ± SD. **P < 0.01, ***p < 0.001, when in contrast with the indicated group

We further estimated the value of DNAJC19 as a potential antitumor target. We used A549 cells treated with Lv-shRNA-DNAJC19 or Lv-shRNA-Control to establish an animal model. In the xenograft mouse model, compared with the negative control Lv-shRNA-Control group (shCtrl group), the Lv-shRNA-DNAJC19 group (shDNAJC19 group) had markedly dampened tumor xenograft growth (Fig. 5A), both in terms of tumor weight (P < 0.001) (Fig. 5B) and tumor volume at days 21, 25, and 27 (P < 0.001) and at days 23 and 29 (P < 0.0001) (Fig. 5C). Moreover, we investigated DNAJC19, PI3K and AKT in xenograft samples by IHC. As shown in Fig. 5F, DNAJC19, PI3K and AKT expression levels were lower in the shDNAJC19 group than in the shCtrl group (Fig. 5D). In the tumor metastasis model, fewer average metastatic tumor lesions in each mouse and fewer mice (80%, 4/5) with lung metastasis were observed in the shDNAJC19 group than in the shCtrl group (Fig. 5E, F). Furthermore, we did HE staining to investigate whether the metastasis lesion exist in mice model. As shown in Fig. 5G, in lung tissue we saw the definitely intrapulmonary metastasis lesion in shControl group. Furthermore, we did immunohistochemistry to assess the Ki-67 expression of tumor metastasis samples. Accordingly, ki-67 expressions are strongly high in shControl group than in shDNAJC19 group (Fig. 5H). These results suggest that DNAJC19 facilitates xenograft tumor growth and tumor metastasis by regulating PI3K/AKT signaling and is a new potential anticancer target molecule in NSCLC.

## Discussion

Patients with advanced NSCLC have a poor prognosis and adverse outcomes, including recurrence and metastasis [21, 22]. With the development of tyrosine kinase inhibitors (TKIs) [23, 24] and immune checkpoint inhibitors (ICIs), the clinical outcomes of advanced NSCLC patients have improved considerably. However, most of these patients develop primary or acquired drug resistance, indicating that many driver oncogenes or biological behaviors of NSCLC tumor cells are not fully understood and need to be further characterized. Overall, exploring novel driver oncogenes is urgently needed to overcome drug resistance and to identify potential therapeutic targets in NSCLC.

The protein encoded by the DNAJC19 gene is considered to participate in a complex involved in the ATP-dependent transport of transit peptide-containing proteins from the inner cell membrane to the mitochondrial matrix. DNAJC19, also named translocase of the inner mitochondrial membrane 14 (TIMM14), is responsible for maintaining the integrity of mitochondria and is linked to cardiovascular disease, as well as cancers [25]. Mitochondrial dysfunction due to changes in DNAJC19 is a common phenotype of tumor cells and is supposed to contribute to the initiation, development and progression of many solid cancers [8]. To our knowledge, we are the first to report the protumor property of DNAJC19 and its regulatory mechanisms in NSCLC.

In this study, we found that DNAJC19 was overexpressed in NSCLC carcinoma tissues, which is consistent with a MEXPRESS analysis from the TCGA data base. Further survival analysis indicated that NSCLC patients with enhanced DNAJC19 expression had poor progression-free survival. These data suggest that DNAJC19 may play an important role in NSCLC tumor development and progression. However, the function of DNAJC19 in NSCLC cells was not clear.

Aberrant regulation of DNAJC19 induces mitochondrial dysfunction, which is critical for cell survival [26]. First, we successfully designed shRNA to knock down DNAJC19 in A549 and NCI-H1299 NSCLC cells and found that DNAJC19 knockdown markedly inhibited NSCLC cell growth and metastasis. Notably, RNA-seq analysis verified that the PI3K/AKT signaling pathway was involved in molecular events when A549 cells were treated with shDNAJC19. Moreover, suppression of DNAJC19 also decreased the expression of PI3K, and AKT proteins in A549 cells. Interestingly, the cell growth, viability and migration abilities were rescued in DNAJC19-knockdown A549 cells by overexpressing AKT. Interestingly, the change of MYC, ERK1/2 or Snail was obvious in A549 cells treated with shDNAJC19 as shown in Fig. 4A, so we speculate that PI3K/AKT/MYC [27], PI3K/AKT/ERK [28] and/or PI3K/AKT/Snail [29] pathway is responsible for DNAJC19 mediated proliferation and migration. We will further research the underlying regulatory mechanism of DNAJC19 in NSCLC in future. Well known, PI3K/AKT signaling is crucial in the regulation of cellular growth and metabolism [30]. In NSCLC, the PI3K/AKT pathway has been implicated in both tumorigenesis and the progression of disease, and somatic mutations of PIK3CA and amplifications of PIK3CA are frequently found in patients with NSCLC [31]. Moreover, cancer cell-intrinsic pathways including PI3K mediate immunosuppression in lung cancer [32]**.** AKT is involved in tumor cell proliferation in breast cancer [33], stem cell properties and apoptosis in non-small-cell lung cancer [34] and tumor progression in gastric cancer [35]. Furthermore, the mouse xenograft model showed that tumor size and weight were significantly decreased in the DNAJC19-knockdown group compared with the negative control group. As expected, compared with the shCtrl group, the shDNAJC19 group exhibited fewer lung metastatic lesions. In addition, the expression levels of DNAJC19, PI3K and AKT in xenograft mouse samples were lower in the shDNAJC19 group than in the shCtrl group. These data suggested that PI3K/AKT is an previously unidentified target of DNAJC19.

In brief, we found for the first time that shRNA-mediated repression of DNAJC19 greatly attenuated tumor cell growth and metastasis by regulating PI3K/AKT signaling, providing a novel therapeutic target for NSCLC patients.

## Conclusion

We reported that high expression of DNAJC19 protein was associated with poor prognosis in NSCLC patients. We successfully designed shRNA to especially and patently inhibit DNAJC19 expression and functions. Finally, we found for the first time that shRNA-mediated repression of DNAJC19 greatly attenuated tumor cell growth and intrapulmonary metastasis by regulating PI3K/AKT, highlighting DNAJC19 as a novel therapeutic target for treating NSCLC patients.

## Supplementary Information

**Additional file 1: Figure S1.** The designed shRNA sequence successfully inhibited the protein and mRNA levels of DNAJC19 in A549 and NCI-H1299 cells. A. The protein expressions of DNAJC19 in different NSCLC cells. B, C Constructed recombinant lentiviral vector expressing shRNA against human DNAJC19 gene (Lv-shRNA DNAJC19) by using the linearized vector GV115. D. The cell status after infection with shDNAJC19 or shCtrl. E. The mRNA level of DNAJC19 measured in lung cancer cells treated with shDNAJC19 or shControl by qPCR.

## Data Availability

The datasets used in the current study are available from the corresponding author on reasonable request.

## Abbreviations

NSCLC: Non-small-cell lung cancer
FBS: Fetal bovine serum
IHC: Immunohistochemistry
TKIs: Tyrosine kinase inhibitors
ICIs: Immune checkpoint inhibitors
PD-1/PD-L1: Programmed death-1/programmed death-ligand 1
EGFR: Epidermal growth factor receptor
ALK: Anaplastic lymphoma kinase
ROS1: ROS proto-oncogene 1
OS: Overall survival
PFS: Progression-free survival
shRNAs: Small hairpin RNAs
