# Supplementary material for: Targeting DNAJC19 overcomes tumor growth and lung metastasis in NSCLC by regulating PI3K/AKT signaling
Source: Cancer Cell Int. 2021 Jul 3;21:338. doi: 10.1186/s12935-021-02054-z (PMC8254338; doi:10.1186/s12935-021-02054-z)
Supplement: Supplementary file 1 — Additional file 1: Figure S1. The designed shRNA sequence successfully inhibited the protein and mRNA levels of DNAJC19 in A549 and NCI-H1299 cells. A. The protein expressions of DNAJC19 in different NSCLC cells. B, C Constructed recombinant lentiviral vector expressing shRNA against human DNAJC19 gene (Lv-shRNA DNAJC19) by using the linearized vector GV115. D. The cell status after infection with shDNAJC19 or shCtrl. E. The mRNA level of DNAJC19 measured in lung cancer cells treated with shDNAJC19 or shControl by qPCR. [file 12935_2021_2054_MOESM1_ESM.pptx]

## Slide 1
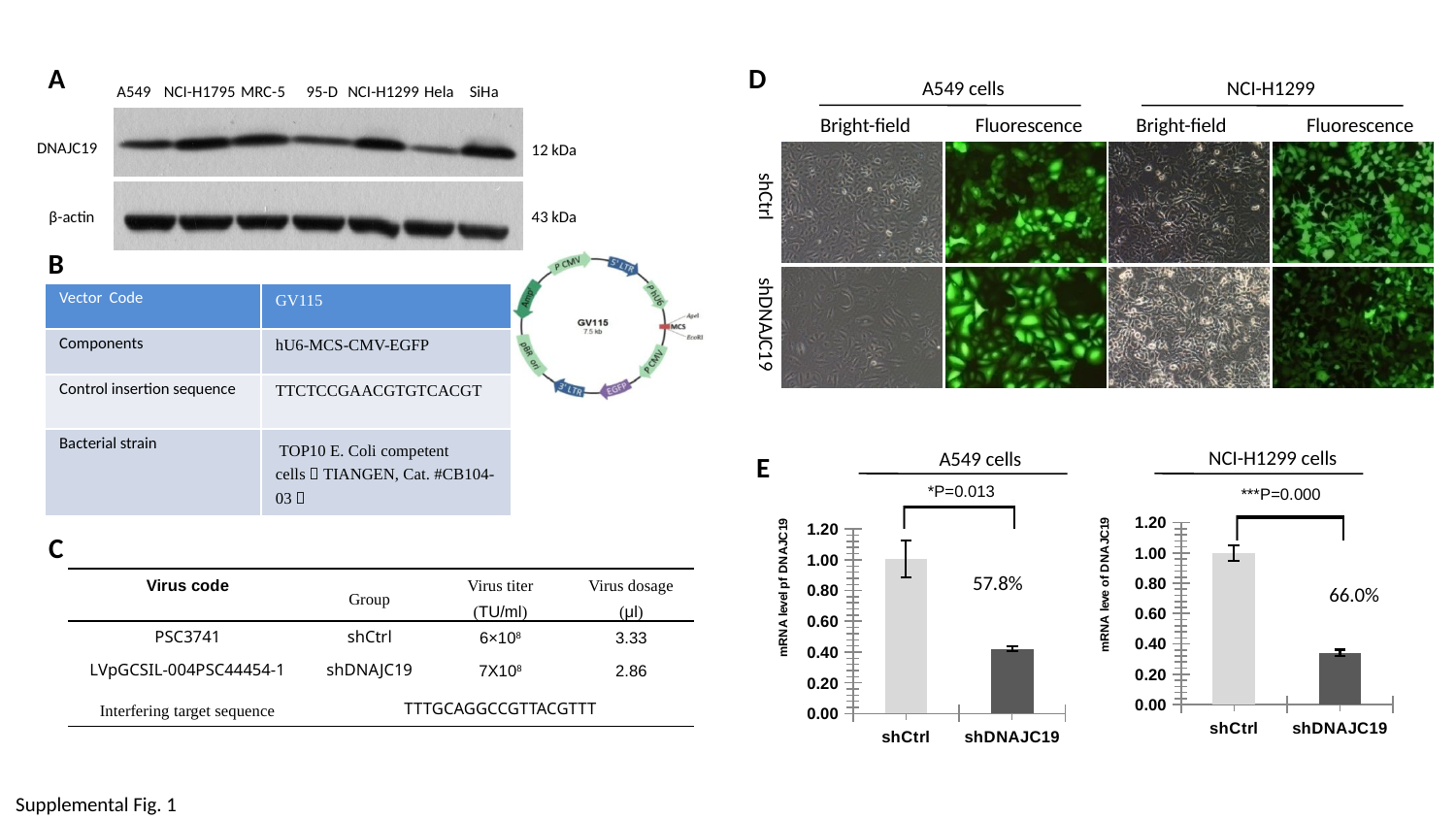

A
D
A549 cells
NCI-H1299
A549
NCI-H1795
MRC-5
95-D
 NCI-H1299
Hela
SiHa
Bright-field
Fluorescence
Bright-field
Fluorescence
DNAJC19
12 kDa
shCtrl
β-actin
43 kDa
B
| Vector Code | GV115 |
| --- | --- |
| Components | hU6-MCS-CMV-EGFP |
| Control insertion sequence | TTCTCCGAACGTGTCACGT |
| Bacterial strain | TOP10 E. Coli competent cells（TIANGEN, Cat. #CB104-03） |
shDNAJC19
### Chart
| Category | Average
(2-ΔΔCt) |
|---|---|
| shCtrl | 1.000877334029568 |
| shDNAJC19 | 0.34036977686507497 |***P=0.000
### Chart
| Category | Average
(2-ΔΔCt) |
|---|---|
| shCtrl | 1.0047397333701438 |
| shDNAJC19 | 0.4216907233675661 |*P=0.013
NCI-H1299 cells
A549 cells
E
57.8%
66.0%
C
| Virus code | Group | Virus titer (TU/ml) | Virus dosage (μl) |
| --- | --- | --- | --- |
| PSC3741 | shCtrl | 6×10^8^ | 3.33 |
| LVpGCSIL-004PSC44454-1 | shDNAJC19 | 7X108 | 2.86 |
| Interfering target sequence | TTTGCAGGCCGTTACGTTT | | |
Supplemental Fig. 1
